# Mature Cystic Teratoma Hiding in the Retroperitoneum of an Adult

**DOI:** 10.1155/2018/8313261

**Published:** 2018-02-18

**Authors:** Yardesh Singh, Shamir O. Cawich, Thivy Kuruvilla, Sidiyq Mohammed, Ammiel Arra

**Affiliations:** Department of Clinical Surgical Sciences, University of the West Indies, St. Augustine Campus, St. Augustine, Trinidad and Tobago

## Abstract

We report a rare case of a mature cystic teratoma found in the retroperitoneum of a 28-year-old woman with vague symptomatology. We review the radiologic and pathologic features of this rare lesion.

## 1. Introduction

Teratomas are nonseminomatous germ cell tumours that arise from abnormal development of pluripotent embryonal germ cells [1]. The retroperitoneum is a rare location for these lesions, with only few primary retroperitoneal teratomas being reported [2–8]. We report an unusual case of a retroperitoneal mature cystic teratoma.

## 2. Presentation of a Case

A 28-year-old woman presented with a vague history of right upper quadrant fullness, vomiting, and worsening upper abdominal pain for eight-week duration. There were no other associated symptoms present.

Physical examination findings were limited to the abdomen. A firm, immobile, nontender mass was palpable in the right upper quadrant. Laboratory investigations revealed a mildly elevated CEA of 7.3 ng/ml (normal < 4.5 ng/ml). The remaining blood investigations were normal: haemoglobin was 13.8 g/dl (normal range 12–14.5 g/dl); CA19-9 was 10 IU/ml (normal < 37 IU/ml), CA125 was 6.95 U/ml (normal value < 35 U/ml), 6 AM cortisol was 11.6 ug/dl (normal 10–20 ug/dl), and AFP was 8 ng/ml (normal value < 10 ng/ml).

**Figure 1:**
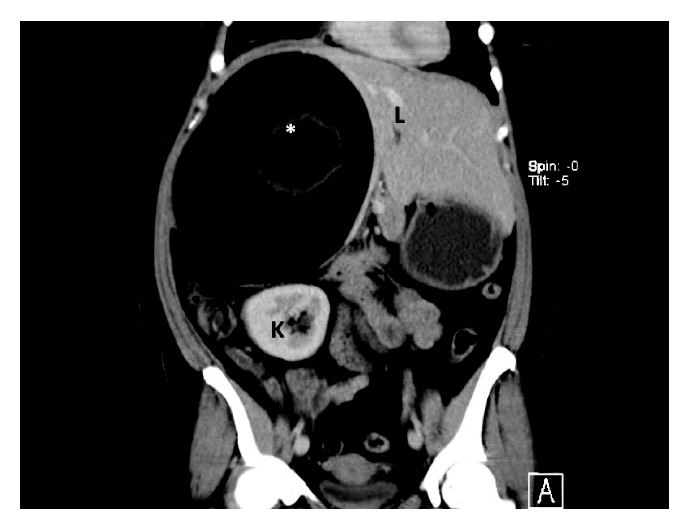
CT scan of the abdomen (coronal view) demonstrating a well-circumscribed cystic mass, with solid components (asterisk), occupying the entire right upper quadrant of the abdomen. The mass measures 19.0 × 19.7 × 13.8 cm in its maximal and displaces the right kidney (K) inferiorly and liver (L) anteromedially.

**Figure 2:**
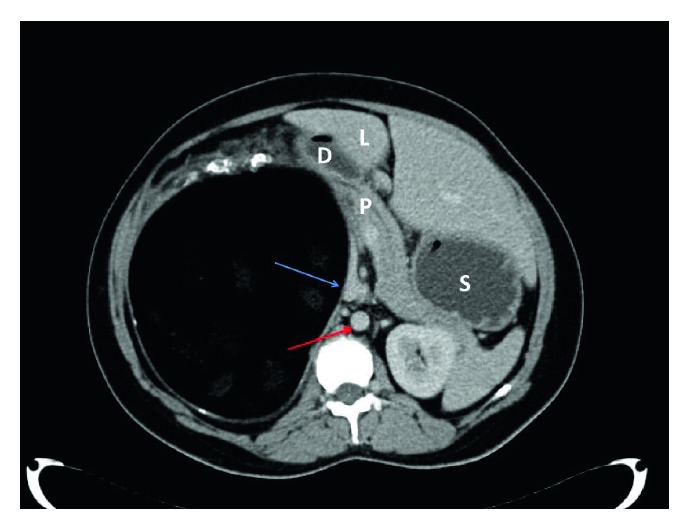
CT scan of the abdomen (axial view) demonstrating the complex cystic mass that appears to arise from the retroperitoneum and displaces the left liver (L), pancreatic head (P), duodenum (D), and inferior vena cava (blue arrow) anteromedially. For orientation, the stomach (S) and aorta (red arrow) are labeled.

Contrast-enhanced computerized tomography (CT) scans revealed a well-circumscribed cystic mass occupying the entire right upper quadrant of the abdomen (Figure 1). The mass measured 19.0 × 19.7 × 13.8 cm in its maximal diameter and contained a central heterogeneous material that was similar to the density of fat. The mass appeared to arise from the retroperitoneum and displaced the liver, pancreatic head, duodenum, and inferior vena cava anteromedially (Figure 2). The right kidney and small bowel were displaced inferiorly. There were well-defined tissue planes separating the mass from surrounding structures, and no metastatic lesions were noted in the chest, abdomen, or pelvis.

The lesion was deemed resectable. Therefore, she was prepared for anaesthesia and taken for exploratory laparotomy. At operation, a large cystic mass was encountered within the retroperitoneum. Thorough exploration of the general abdominal cavity revealed no evidence of metastatic disease or ascites. The mass could be dissected from the diaphragm, liver, pancreatic head, duodenum, transverse mesocolon, and vena cava. There was a tissue plane separating the mass from the right kidney, but there was not a distinct plane that separated it from the right adrenal gland. Therefore, the right adrenal gland was excised en bloc with the mass.

**Figure 3:**
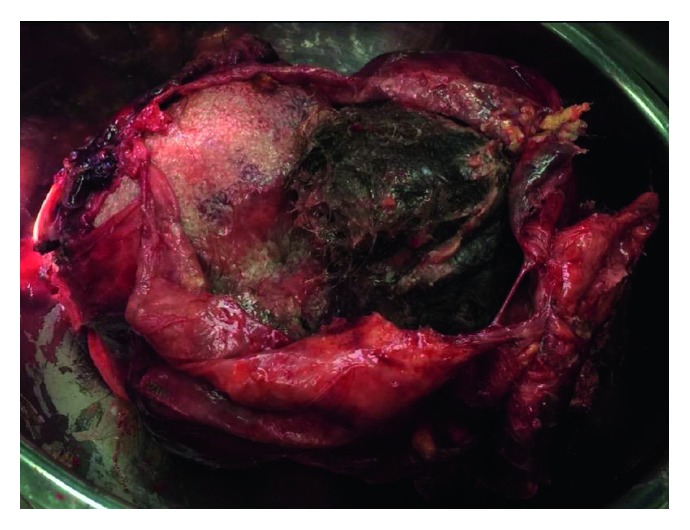
The excised cystic mass has been opened in longitudinal section. Thick yellow fluid has been evacuated from the cyst, leaving hair and sebum visible and small deposits of the bone and cartilage visible within the sac.

**Figure 4:**
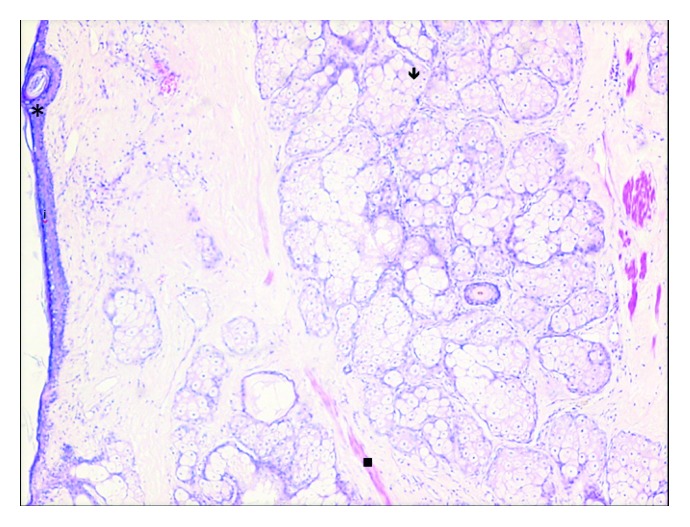
Histologic sections at low power reveal ectodermal components of the skin, sebaceous glands (↓), and smooth muscle (■) within the cyst wall. Mature adipocytes are present elsewhere in the photomicrograph. ^∗^Cross section through the smooth muscle.

A heterogeneous mass was removed and sent for histopathologic evaluation. The cyst wall was 4 mm thick. On sectioning, the cyst contained thick yellow fluid, hair, and sebum (Figure 3). The mass contained predominantly fatty tissues, with small deposits of bone, cartilage, and areas of calcification. Histologic sections revealed ectodermal components of skin and hair adnexal structures within the cyst wall (Figure 4). Mature adipocytes and lymph nodes could be identified. There were no immature elements detected and no evidence of malignancy identified.

This patient had an uneventful postoperative recovery period. She was discharged at day five after operation and has remained well two years after resection.

## 3. Discussion

Teratomas are nonseminomatous germ cell tumours that arise from pluripotent embryonal germ cells [1]. Germ cells develop during embryogenesis and usually descend along a midline path into the pelvis to form ovarian cells, or into the scrotum, forming testicular cells [2, 5]. If these cells fail to migrate along the urogenital ridge, germ cells may be deposited in extragonadal sites and are at risk for neoplastic conversion [2, 5]. Therefore, teratomas can occur in the ovaries, testes, anterior mediastinum, retroperitoneum, and cranium, in order of decreasing frequency [2–8].

Teratomas in the retroperitoneum are uncommon. They constitute between 1% [2] and 10% [6] of all primary retroperitoneal tumours and are usually encountered in children within the first six months of life [2–6]. Primary retroperitoneal teratomas in adults are rare, with only a handful of cases reported in medical literature [2–8]. They are twice as common in females compared to males [3] and are usually encountered on the left, near the upper pole of the left kidney [3]. Therefore, this was a very unusual case in terms of the patient's demographics and the location in the retroperitoneum over the right kidney.

In the retroperitoneum, these lesions usually remain asymptomatic until they have attained a large size, thus producing compressive symptoms or mass effect. There are no pathognomonic features of a retroperitoneal teratoma on plain radiographs, although they may demonstrate calcification. Ultrasound is a useful first investigation because it can differentiate between solid and cystic components and may be able to detect calcification, teeth, and/or bone [5]. But the diagnosis ultimately requires computed tomography scanning as it provides better resolution of the soft tissues and it can identify features suggestive of malignancy [5–7]. It usually appears as a complex mass with a well-circumscribed fluid component, containing adipose tissue and calcification [8]. The presence of hypoattenuating fat within the cyst is considered highly suggestive of cystic teratoma [8]. With the presence of calcifications in the cyst wall, cystic teratoma is even more likely [8]. MRI has also been used as these lesions have a high fat content and appear hyperintense on T1-weighted images [7].

Tumour markers, such as alpha-fetoprotein (AFP), carcinoembryonic antigen (CEA), CA19-9, and CA125, are routinely evaluated in patients with suspected teratomas. We reviewed four large retrospective studies that evaluated tumour marker levels in a total of 970 patients with mature cystic teratomas [9–12]. Cumulative analysis revealed that the commonest markers to be elevated in these patients were CA19-9 in 42% (409/970) of confirmed cases and CA125 in 16% (128/813) of confirmed cases. All authors uniformly reported that these tumour markers were limited for diagnostic purposes [9–12]. However, there was a significant correlation between elevated CA19-9 and tumour size [9–12], presence of adhesions [11], and torsion rates when they arise at the ovaries [11]. However, there was no other correlation with clinicopathologic characteristics. Nevertheless, routine tumour marker assays are advocated as the results could be used for postoperative tumour surveillance [9–12].

Although some authors have reported elevated CEA levels in patients with malignant conversion in retroperitoneal teratomas [13, 14], the relationship is not well established. In our patient, there was a mildly elevated CEA, but no obvious malignant features were identified. This was not surprising, considering that the absence of mature tissues [3], absence of sebum [4], and occurrence in childhood years [2, 15] have been reported to be predictors of malignant change.

Malignant lesions are notoriously resistant to systemic chemotherapy and irradiation [2]. Therefore, aggressive surgical resection with oncologically clear margins is advocated for treatment of these patients. There is near 100% five-year survival after complete excision [16]. Systemic chemotherapy and irradiation are relegated only to patients with malignant features on histopathologic evaluation [2].

## 4. Conclusion

Mature retroperitoneal teratomas are rare lesions. They are typically asymptomatic, although they may be diagnosed preoperatively by imaging with CT and MRI. Surgical resection is the mainstay of treatment for mature retroperitoneal teratomas, yielding near 100% 5-year survival when margins are oncologically clear.
